# Life in a Droplet: Microbial Ecology in Microscopic Surface Wetness

**DOI:** 10.3389/fmicb.2021.655459

**Published:** 2021-04-13

**Authors:** Tomer Orevi, Nadav Kashtan

**Affiliations:** Department of Plant Pathology and Microbiology, Robert H. Smith Faculty of Agriculture, Food, and Environment, Institute of Environmental Sciences, Hebrew University, Rehovot, Israel

**Keywords:** plant microbiome, soil microbiology, phyllosphere, hydration conditions, microscopic surface wetness, deliquescence, wet-dry cycles

## Abstract

While many natural and artificial surfaces may appear dry, they are in fact covered by thin liquid films and microdroplets invisible to the naked eye known as microscopic surface wetness (MSW). Central to the formation and the retention of MSW are the deliquescent properties of hygroscopic salts that prevent complete drying of wet surfaces or that drive the absorption of water until dissolution when the relative humidity is above a salt-specific level. As salts are ubiquitous, MSW occurs in many microbial habitats, such as soil, rocks, plant leaf, and root surfaces, the built environment, and human and animal skin. While key properties of MSW, including very high salinity and segregation into droplets, greatly affect microbial life therein, it has been scarcely studied, and systematic studies are only in their beginnings. Based on recent findings, we propose that the harsh micro-environment that MSW imposes, which is very different from bulk liquid, affects key aspects of bacterial ecology including survival traits, antibiotic response, competition, motility, communication, and exchange of genetic material. Further research is required to uncover the fundamental principles that govern microbial life and ecology in MSW. Such research will require multidisciplinary science cutting across biology, physics, and chemistry, while incorporating approaches from microbiology, genomics, microscopy, and computational modeling. The results of such research will be critical to understand microbial ecology in vast terrestrial habitats, affecting global biogeochemical cycles, as well as plant, animal, and human health.

## Introduction

**Figure 1:**
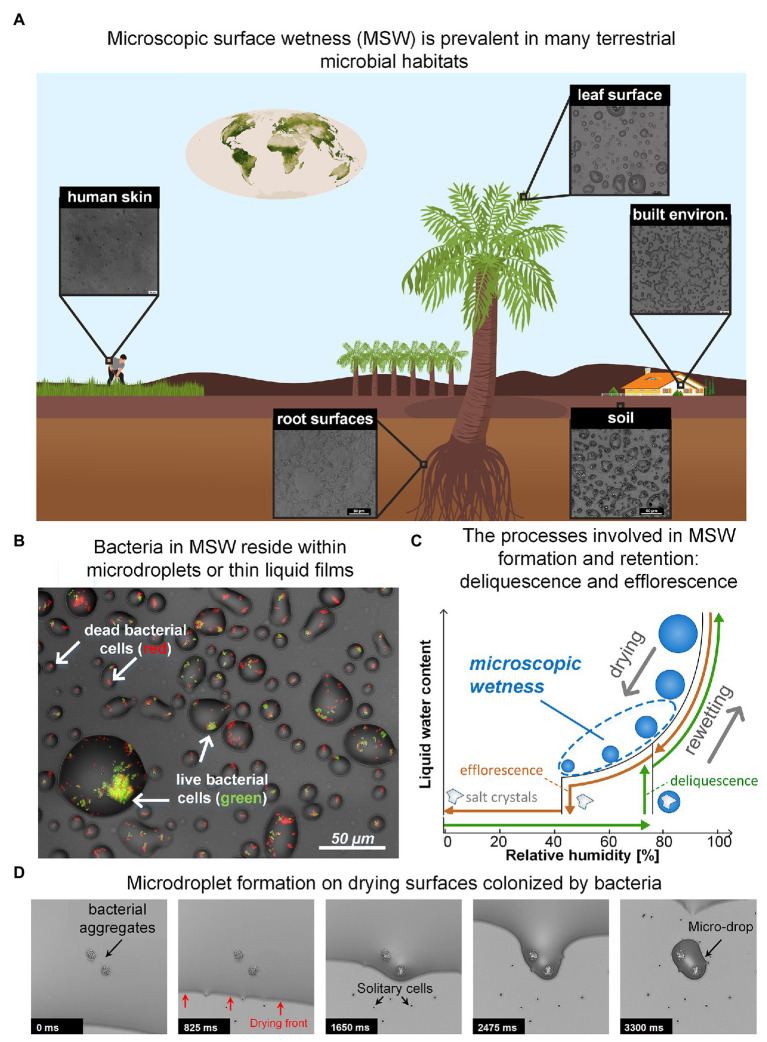
**(A)** Microscopic surface wetness (MSW) is predicted to exist in various terrestrial microbial habitats. Insets show MSW images that result from natural samples that were dried on glass surfaces at 60–70% RH. The chemical compounds necessary for MSW formation, e.g., deliquescent compounds, are abundant in most terrestrial microbial habitats. **(B)** Bacteria in MSW. Bacteria in larger microdroplets typically exhibit higher survival. **(C)** A hysteresis diagram describing the underlying processes driving the formation and retention of MSW. Drying of a solution that contains deliquescent compounds leads to the formation of MSW when the RH is above the compounds’ efflorescence point (brown path). In the opposite direction – i.e., rewetting – solid crystalline deliquescent compounds turn into MSW when the RH is above their point of deliquescence (green path). Typically, the efflorescence point is lower than the deliquescence point of a specific salt. **(D)** Time lapse showing how microdroplets are formed around bacteria aggregates and cells on a drying surface. (Panel **A**: Earth vegetation map is from NASA website; Panels **B** and **D**: images adapted from Grinberg et al., 2019b).

While water is essential to life, a large portion of microbial life occurs on surfaces that are not constantly saturated with water. Remarkably, though many of these surfaces appear dry, they are often covered by thin liquid films and micrometer-sized droplets, invisible to the naked eye, which we term microscopic surface wetness (MSW; Figures 1A,B; Burkhardt and Hunsche, 2013; Grinberg et al., 2019b). Key to the formation and retention of microscopic surface wetness is the presence of deliquescent substances – mostly highly hygroscopic salts – that absorb moisture from the air until they dissolve-in and form a liquid solution (Martin, 2000; Wise et al., 2008; Mauer and Taylor, 2010). Thus, residual deposits of deliquescent compounds that cover a surface turn into, or are retained as, MSW when the relative humidity (RH) exceeds a specific point. As deliquescent substances, such as salts, are ubiquitous, MSW likely occurs in many microbial habitats including leaf and root surfaces (Burkhardt and Eiden, 1994; Burkhardt et al., 2001; Burkhardt, 2010; Burkhardt and Hunsche, 2013; Katata and Held, 2020), soil and rock surfaces (Wierzchos et al., 2012; Davila et al., 2013; Dai et al., 2016; Sato and Hattanji, 2018), the built environment (Trechsel, 1994; Schwartz-Narbonne and Donaldson, 2019), and probably even on our skin (Patrick et al., 1997) (Figure 1A). Hence, microscopic surface wetness is a common permanent or transient hydration state occurring in diverse important terrestrial microbial habitats.

MSW’s formation can result from both drying and rewetting of surfaces. Drying of a solution that contains soluble deliquescent compounds leads to the formation of MSW when the RH is above the compounds’ efflorescence point – the point where salt crystallization occurs and residual water evaporates (Mauer and Taylor, 2010; Figures 1B,C). In the opposite direction (i.e., rewetting), solid crystalline deliquescent compounds dissolve-in and form a solution (turning into MSW) when the RH is above their deliquescence point (Mauer and Taylor, 2010; Figure 1C). In both directions, the result is a stable, highly-concentrated solution with micrometer dimensions in the form of thin liquid films and microdroplets that cover the surface (Figure 1B). MSW’s inherent properties – exceptionally high salt concentrations, extremely small volumes, and segregation into droplets – create a heterogeneous micro-environment at the microscale resolution that differs significantly from bulk liquid solutions (Rubasinghege and Grassian, 2013; Vejerano and Marr, 2018; Campbell et al., 2019). Consequently, MSW affects many aspects of microbial life therein.

One of the reasons why MSW has not been thoroughly studied in the context of microbial ecology is that most microscopy-based research in experimental microbiology is conventionally conducted in water-saturated systems, or on dry agar plates, both of which tend to modify the environmental conditions that create MSW on a specimen, namely humidity. As a result, traditional methods have barely exposed MSW, which was therefore largely overlooked.

Importantly, there are major distinctions between MSW and the more well-studied microscopic wetness in porous media (e.g., in soil; Brooks and Corey, 1966; Laurindo and Prat, 1998; Prat, 2007; Lehmann et al., 2008; Dullien, 2012). The focal difference is that MSW results mainly from deliquescent properties of solutes, and not from capillary forces (Laurindo and Prat, 1996; Tuller et al., 1999). In addition, MSW occurs on smooth or rough surfaces and is not restricted to pores. Last, MSW often has a direct interface with the atmosphere, which is limited in porous media (Shokri et al., 2009). These differences are reflected in key properties of MSW, which are important to bacteria and fungi, including very high salinity, the balance between the matric and solute water potentials, and possibly other properties.

While the impact of porous media on soil microbial ecology has been explored (Young and Crawford, 2004; Or et al., 2007b; Dechesne et al., 2008, 2010; Hinsinger et al., 2009; Carson et al., 2010; Ruamps et al., 2011; Vos et al., 2013; Raynaud and Nunan, 2014; Tecon and Or, 2017; Baveye et al., 2018; Portell et al., 2018), studies on microorganisms in MSW environments are few, and mostly aimed at exploring the feasibility of life under water limitation and the capability of deliquescent wetness to support microbial life. The pioneers in research about life in MSW environments were astrobiologists and microbiologists interested in extreme environments. The feasibility of life in deliquescent wetness, in salt brines, and under low water potential has been explored, mainly focusing on hyper-arid deserts and extra-terrestrial environments. Intermittent deliquescent microscopic wetness was shown to support microbial life and photosynthesis in the Atacama Desert (Navarro-González et al., 2003; Warren-Rhodes et al., 2006; Davila et al., 2008, 2010, 2013; Gómez-Silva, 2018). Several bacterial species were shown to have the ability to survive in deliquescent salts of Mars-analog environment (Jänchen et al., 2016; Nuding et al., 2017; Heinz et al., 2019; Stevens et al., 2019; Hallsworth, 2020). The limits of water activity to support life have also been explored (Stevenson et al., 2015a,b). Interestingly, deliquescent wetness on leaves of salt-excreting plants was suggested to protect phyllospheric microorganisms from complete desiccation (Simon et al., 1994). These ground-breaking studies, from various fields, provided early insights into the ability of microorganisms to survive in MSW. Yet, systematic studies on the various ways that MSW conditions affect microbial life and ecology are only in their beginning. As MSW is likely prevalent (often transiently) in diverse vast terrestrial microbial habitats, and not necessarily only in what are considered as extreme habitats, closing this knowledge gap is crucial to understanding microbial ecology in these immense ecosystems.

In a recent study, we suggested that MSW formation from the deliquescence of hygroscopic aerosols – prevalent on leaves (Tang, 1980; Tang and Munkelwitz, 1993; Burkhardt et al., 2001; Pöschl, 2005) – might explain how bacteria survive daytime dryness on leaves (Grinberg et al., 2019b; Tecon, 2019). Using an *in vitro* experimental system developed in our lab, we revealed that bacterial cells – aggregates in particular – retained a hydrated micro-environment in the form of stable microscopic droplets (of tens of μms in diameter) on drying surfaces at moderate humidity, due to a combination of capillary pinning and solutes’ deliquescent properties (Grinberg et al., 2019b). Similar findings were observed for 13 bacterial species from diverse habitats, including Gram-negative and Gram-positive bacteria (Grinberg et al., 2019b). These findings corroborate previous observations reporting soft, liquid-like substances wrapped around bacterial cells post drying, suggested to form due to deliquescence of solute components (Méndez-Vilas et al., 2011), as well as observations reporting the retention of liquid films around fungal hyphae (Stabentheiner et al., 2010). Intriguingly, we further revealed that larger droplets formed around larger bacterial aggregates and that cell survival increased with droplet size (Figures 1B,D). These findings exposed new reciprocity between microbiology and physics, wherein the physical properties of water enable groups of cells to collectively retain more water around them, in turn increasing their own survival. The net outcome is a strong coupling between bacterial self-organization on surfaces, MSW, and cell survival (Grinberg et al., 2019b).

Our experimental system enabled us to better understand how bacterial colonization affects MSW formation on surfaces. Retention of droplets around aggregates and cells, through pinning of the liquid-air interface, was clearly evident (Figure 1D). This pinning is due to capillary forces associated with surface roughness produced by the presence of bacteria (Herminghaus et al., 2008; Bonn et al., 2009). This phenomenon indicates that aggregate size (and possibly other properties) determines droplet size. The overall survival rate of cells was low, indicating that MSW is indeed stressful for bacteria, at least in part due to low water potential (with a significant negative contribution of osmotic potential, which is inherent to MSW that forms from deliquescent properties of solutes). Notably, cell survival was higher in larger droplets (Figure 1B; Grinberg et al., 2019b). While it was known that bacterial aggregates and biofilms provide protection from desiccation, this was mostly attributed to extracellular polymeric substances (EPS) and their function as a hydrogel (Roberson and Firestone, 1992; Ophir and Gutnick, 1994; Chang et al., 2007; Or et al., 2007a). Our results reveal a new, additional function of bacterial aggregation: improving hydration by retaining large droplets which in turn, increase cell survival in environments frequently exposed to drying (Grinberg et al., 2019b).

**Figure 2:**
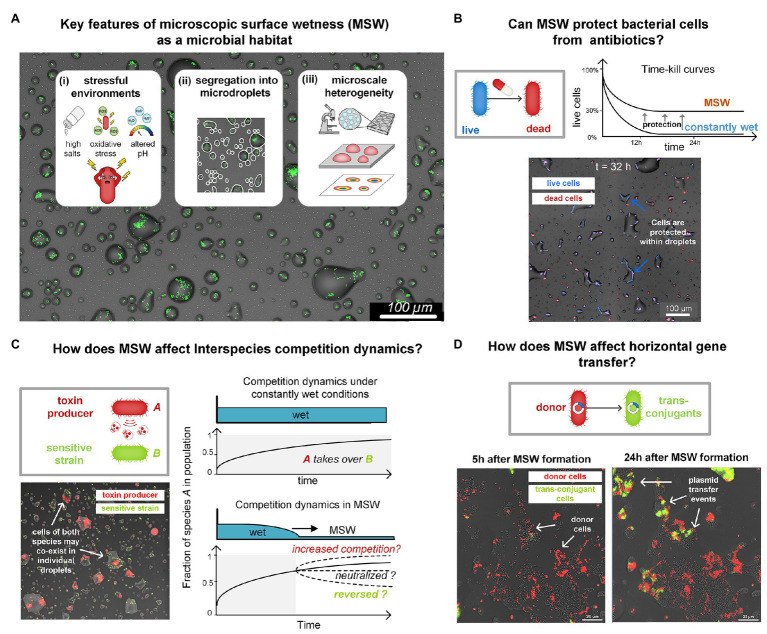
**(A)** Key features of MSW as a microbial habitat. **(B)** Inherent properties of MSW, including very high salt concentrations and slow cell-growth protect bacteria from various antibiotics. In a recent study, we showed such protection of *Escherichia coli* from major antibiotic classes (e.g., beta-lactams; Beizman-Magen et al., 2020). The schematic illustration shows killing curves of *E. coli* in response to Ampicillin under constantly wet conditions and on a drying surface with MSW. Survival of *E. coli* was more than two orders of magnitude higher in MSW than in constantly wet condition. The micrograph shows cells that survive long periods of exposure to Ampicillin under MSW conditions. Live cells express BFP (blue) and dead cells are marked with propidium iodide (red). **(C)** Key features of MSW mentioned in **(A)** are predicted to impact interspecies competition dynamics. Interference competition in wet-dry cycles and MSW might, for example, be neutralized due to antibiotic protection, as shown in **(B)**. Preliminary results show a surface section colonized by two species: *Pseudomonas fluorescens* A506 (a bio-control agent) and *Pseudomonas syringae B728a* (Plant Pathogen). *Pf* A506 is known to antagonize *Ps* B728a under wet (saturated) conditions. Although under MSW many droplets contain cells of both strains, this antagonism seems to be neutralized. This indicates that competition dynamics under water-saturated conditions and those under MSW can differ widely. Competition could potentially increase, neutralize, or even reverse. **(D)** How does MSW affect horizontal gene transfer? Formation of MSW confine bacteria cells in droplets or films, possibly promoting cell-to-cell physical contact. Yet, the altered physicochemical conditions and physiological state of cells, in comparison to water-saturated environments, may affect rates of genetic exchange, e.g., plasmid transfer rates. The images show a population of *Pseudomonas putida* KT2440 cells that were engineered to report conjugation events. Plasmid Donors (red) and Recipients (gray) were exposed to a gradual drying episode. As can be seen, shortly after MSW formation (*t* = 5 h), there were only few conjugation events, visible by the emergence of green fluorescent cells. However, at *t* = 24 h, many more conjugation events occurred, indicated by the number of green trans-conjugant cells.

However, while possibly protecting from desiccation, MSW is a harsh micro-environment different from bulk liquid (Figure 2A). The physicochemical properties associated with MSW, such as salinity, pH, and reactive oxygenic species (ROS), and its microscale heterogeneity (Rubasinghege and Grassian, 2013; Vejerano and Marr, 2018; Campbell et al., 2019), necessarily impose severe stresses on cells (Potts, 1994; Xie et al., 2006; Runkel et al., 2013; Alsved et al., 2018; Figure 2A). An investigation of bacterial life in MSW must identify the challenges that this micro-environment poses as well as the unique adaptations that enable bacteria to function in such harsh microenvironments. We thus hypothesize that MSW affects key aspects of bacterial life and ecology besides cell survival, including tolerance to antibiotics, competition, cell-to-cell interactions, communication, motility, and exchange of genetic material (Figures 2B–D), as demonstrated for soil and other porous media (Massoudieh et al., 2007; Dechesne et al., 2010; Tecon et al., 2018).

In the following sections, we outline ongoing research and future directions that we believe are essential to advancing our understanding of microbial ecology under MSW conditions. While we focus in the following sections on bacterial ecology, these directions are relevant for fungi as well.

## Prevalence of MSW in Natural Microbial Habitats

To begin to appreciate how common MSW is and what general properties are shared by various “real-world” chemical environments, there is a need to survey the relevant habitats, wherein MSW is predicted to occur, permanently or transiently. Observation of MSW in natural settings poses a technological challenge because they require microscopic inspection *in situ* at ambient RH. So far, such direct observation methods are unavailable (Burkhardt and Hunsche, 2013). Environmental scanning electron microscope (ESEM) allow the inspection of wet samples, yet under controlled non-ambient conditions (Stabentheiner et al., 2010; Burkhardt et al., 2012). An alternative approach is to study environmental samples under controlled conditions in the lab. In a very initial effort in this direction, we tested environmental samples collected from various terrestrial microbial habitats, under controlled RH levels. Importantly, MSW was observed in a variety of samples that were exposed to moderate RH (60–70%). MSW resulting from soil extracts, natural leaf and root washes, human sweat, and surfaces sprayed with disinfectants were similar (visually) to that from experiments with inoculated bacteria in minimal synthetic medium (Grinberg et al., 2019b; Figures 1A,B). Retention of droplets and films were, for example, observed around fungal hyphae, yeast, and bacteria from samples washed from leaf surfaces (see, e.g., Figure 4 in Grinberg et al., 2019b). This simple indirect method indicates that deliquescent compounds are common in diverse habitats and inevitably, will manifest in the form of MSW when the ambient humidity is above the efflorescence or deliquescence points (while drying or rewetting, respectively). For most of these samples a moderate RH of 60–70%, that is known to be realized, at least transiently, in many of these environments, will suffice. In addition, these observations suggest that unifying principles govern the formation and retention of MSW across many different microbial habitats. Obviously, one can expect to find differences between MSW in different habitats, which result from variation in surface roughness, chemistry of natural solutions, microbial activity, and differences in RH levels and dynamics between habitats.

## Characterization of the Fundamental Physical, Chemical, and Biological Dimensions of MSW As a Microbial Habitat At Microscale Resolution

Well-founded knowledge of the conditions that bacteria confront in MSW surroundings, as well as deep understanding of the processes and forces that form and shape MSW, is currently missing. Investigation of MSW forming from both synthetic and environmental samples from microbial habitats, wherein MSW is predicted to frequently occur, will serve to elucidate the general properties of MSW. Microscopy-based experimental platforms similar to the one described earlier (Grinberg et al., 2019b), that enable the quantitative study of bacterial life in MSW, through live-imaging at the single-cell level, with micrometer-scale resolution will be useful for such investigations. The development of tools to map the MSW environment at the microscale, including, e.g., fluorescence-based micro-sensors (Johnson and Spence, 2010) and bacterial bio-reporters (Leveau and Lindow, 2002), will enable measuring variables, such as salts, pH, and ROS, at micrometer resolution. These methodologies can be further used to generate a multi-layer, microscale-resolution, spatiotemporal map of MSW microenvironments containing both physicochemical properties of the system and bacterial organization, physiological state, and survival at single-cell resolution (Figure 2A). This information is crucial for understanding the environmental context that microorganisms confront, respond to, and evolve under.

## Identifying and Analyzing Bacterial Traits That Increase Survival in MSW

Surviving through periods of MSW conditions is likely challenging for bacteria (Grinberg et al., 2019b). Consequently, bacteria must have evolved various traits and strategies to cope with MSW. What genes contribute the most to fitness in MSW and how? What phenotypes and traits are associated with these genes? These might include physiological traits of individual cells, such as assembly of ion pumps or production of cytoplasmic osmolytes (Record et al., 1998; Wood, 2015). Collective traits, which lend benefit when groups or populations of cells act together (usually through cooperation), may include: (i) behavioral traits such as aggregation, that may depend upon a set of cellular apparatuses (e.g., pili; Li et al., 2007), on sensing, and on lifestyle decision-making (Ho et al., 2017) and (ii) secretion of public goods such as EPS components, extracellular enzymes, and bio-surfactants (Cordero et al., 2012; Allen et al., 2013; Hernandez and Lindow, 2019). A promising methodology for studying the contribution of genes to fitness and survival in MSW is the rapidly advancing TnSeq (Van Opijnen et al., 2009) that builds upon the construction of a random transposon mutant library and whole-genome screen. We predict that genes found to be associated with a positive contribution to fitness in MSW will include both individual and collective traits. Further work will be required for in-depth study of the traits associated with these genes and how they contribute to fitness in MSW. Of special interest are traits involved in lifestyle strategies and decision-making, which we predict are of great importance in environments undergoing wet-dry cycles with MSW periods, with alternating selection pressures between growth (when wet) and survival (when dry).

## Uncovering the Complex Two-Way Interactions Between Microbes and the Physical Environment of MSW

We anticipate that collective traits will be vital for survival under MSW. Our recent work (Grinberg et al., 2019b) highlights a strong coupling between bacterial self-organization on surfaces, MSW, and bacterial survival. Further work is required to better understand the link between beneficial collective traits and fitness in MSW. For example, bacterial organization resulting from different lifestyles, such as planktonic vs. biofilm, solitary vs. aggregated, and various aggregation forms, e.g., surface attached, suspended flocs, and floating pellicles, are expected to affect MSW formation, which in turn likely affect cells’ fitness. We should also better understand how bacterial activities affect the system. For example, bacteria are able to modify surface wettability by collective secretion of bio-surfactants (Burch et al., 2016), yet this has not been studied *vis-à-vis* MSW. Finally, the impact of surface roughness and shape, at the microscale, should be explored with respect to microbial organization, MSW and the interplay between them (Doan et al., 2020). As in many other complex microbial systems, gaining mechanistic understanding of this system requires a combination of experiments and computational modeling approaches.

## Computational Modeling and Simulations To Complement Experimental Studies on Bacteria in MSW Environments

The heterogeneous nature of the MSW state at the microscale (Figure 2A), unlike well-mixed saturated environments, demands appropriate approaches to investigation. To improve our understanding of the complex MSW environment as a microbial habitat, a combination of experiments alongside modeling is required. Individual-based models (Grimm and Railsback, 2005) have been demonstrated as a powerful research tool to study ecology and evolution of microbial systems (Kreft et al., 2001; Xavier and Foster, 2007; Hellweger and Bucci, 2009; Lardon et al., 2011; Kim and Or, 2016; Jayathilake et al., 2017; Cornell et al., 2019). The importance of the individual-cell as opposed to population-based approaches has been highlighted in microbial ecology (Kreft et al., 2013; Remus-Emsermann et al., 2013; Leveau et al., 2018; Schmidt et al., 2018; Grinberg et al., 2019a). Individual-based models are based on a bottom-up approach that has proved useful for studying how spatiotemporal patterns or properties of interest in a given system emerge from the behavior of individuals, interactions between individuals, and one- or two-way interactions between individuals and their environments. The dynamic and spatially heterogeneous MSW environment, at the microscale, hampers the adequacy of utilizing population-averaged models. We therefore believe that research directions to study microbial ecology in MSW will benefit if complemented by individual-based models. For example, using individual-based modeling approaches can help to better understand how individual-cell behavior affect surface colonization patterns, which then affect microscopic waterscapes, that in turn affect fitness of individual-cells.

## Bacterial Response To Antibiotics Under MSW

A large variety of antibiotic compounds are abundant in microbial habitats, where MSW occurs. In soil, leaf, and root surfaces, major sources of antibiotics are their natural production by microbes and plants (Wells et al., 1982; Williams and Vickers, 1986; Raaijmakers et al., 1997; Forsberg et al., 2012), as well as their release into water and soil by human activity and agricultural practices (Mompelat et al., 2009; Jechalke et al., 2014; Franklin et al., 2016). These antibiotics engage in microbial warfare and competition and consequently affect population dynamics and compositions of microbial communities. Yet, bacterial response to antibiotics on surfaces with permanent or temporary MSW conditions has been scarcely studied.

In a recent study, using *Escherichia coli* as a model species, we revealed that cells are inherently protected from beta-lactam antibiotics under wet-dry cycles with an MSW phase (Beizman-Magen et al., 2020). Bacteria exposed to high concentrations of antibiotics – considerably higher than the minimum inhibitory concentrations (MIC) – exhibited significantly higher survival under a wet-dry cycle with prolonged MSW conditions than they did under constantly wet conditions (Beizman-Magen et al., 2020; Figure 2B). Through a combination of experiments and computational modeling, we were able to identify the mechanisms participating in the observed increased protection: including cross-protection due to high salt concentrations and tolerance induced by slow growth and the physiological state of cells. These findings highlight the fundamental discrepancy between microbial life in MSW and in water-saturated environments.

## Competition and Inter-Species Interactions in MSW

Most microbes do not live in isolation, but rather in complex communities, where many species co-exist, and thus inter-species interactions are prevalent. Competition is likely the most common interaction found in microbial communities (Chao and Levin, 1981; Riley and Gordon, 1999; Foster and Bell, 2012; Cornforth and Foster, 2015; Ghoul and Mitri, 2016; Bauer et al., 2018). Interference competition, for example, facilitated by the production of antimicrobials that are released into the surroundings or injected into neighboring cells (Ma et al., 2014; Bernal et al., 2018), is predicted to be affected by key properties of MSW as a microbial habitat. Segregation of small sub-populations into droplets is expected to impact competition and co-existence (Iwasa and Roughgarden, 1986; Nee and May, 1992; Hanski, 2008; Wang and Or, 2013) and to render inter-species interactions more stochastic in highly diverse communities (Hsu et al., 2019; Figure 2C). The wide range of droplet sizes creates a heterogeneous waterscape with varying local-population sizes and carrying capacities (Remus-Emsermann et al., 2012). Our early findings indicate that the harsh MSW environment likely reduces competition for nutrients while increasing selection for survival. Since wet-dry cycles are common in habitats where MSW occurs, it is reflected by periods of faster growth, migration, and population mixing when wet, followed by segregation and selection for survival when “dry.” In addition, the modified physicochemical properties of MSW may affect cell-to-cell interactions. Finally, the imposed confinement limits dispersal, and thus cell-to-cell contact may be increased (Tecon et al., 2018) and secreted molecules may reach high concentrations.

## Horizontal Gene Transfer in MSW

Understanding how MSW conditions affect horizontal gene transfer is of great importance to microbial ecology and evolution. Conjugal plasmids, for example, constitute an engine for horizontal gene transfer and are main drivers of the spread of antibiotic resistance among bacteria (Levy and Marshall, 2004). Under MSW conditions, cells are confined within droplets or films for prolonged periods, and thus cell-to-cell interactions mediated by physical contact are expected to be high. In a recent study, Tecon and Or showed that (matric) water potential affects plasmid transfer rates in porous media (Tecon et al., 2018). Using reporter conjugal plasmids (Klümper et al., 2015), we observed plasmid transfer events within individual microdroplets (Figure 2D). It is not yet clear, however, whether and how the high salinity of MSW, confinements in microdroplets, and cells’ physiological and metabolic state, affect the exchange of genetic material, and how these transfer rates compare to water-saturated conditions. Interestingly, high conjugation rates were reported on leaf surfaces with clear dependency on the environmental RH. This finding implies that local wetness conditions and MSW modulate horizontal gene transfer rates (Normander et al., 1998; Björklöf et al., 2000).

## Conclusion and Outlook

The current Perspective highlights the importance of studying microbial life and ecology in microscopic surface wetness, and further outlines our view on the essential future research directions. We hypothesize that key features of MSW as a microbial habitat including (1) highly concentrated solutions within droplets and films (2) segregation into extremely small volumes within microdroplets or films and (3) altered cell physiology and slow cell growth, extensively affect central aspects of microbial ecology in environments with permanent or intermittent MSW conditions. The outlined research directions are expected to change how we understand microbial ecology in vast terrestrial habitats, where MSW is expected to commonly occur.

The robustness of MSW formation, in its many forms, suggests that it occurs in a vast range of terrestrial microbial habitats and for prolonged periods of time. We thus believe that these studies on bacterial survival under the extreme environments of hyper-arid deserts, Mars-analog environment, and salt brines, are in fact highly relevant to conditions that bacteria commonly encounter in vast habitats on Earth. Microorganisms (bacteria and fungi) in the largest terrestrial habitats undergoing recurrent wet-dry cycles and MSW, likely experience similar extreme conditions – including exceptionally high salinity and very low water activity – on a daily basis.

Accordingly, further research of this topic would greatly enhance what we know and how we perceive microbial life in large parts of the world around us. As MSW is predicted to be ubiquitous in terrestrial microbial habitats, including plant leaves, soil and rock surfaces, it may change how we understand microbial life in these environments and its effect on global biogeochemical cycles. As MSW likely occurs on plant and animal surfaces, we believe that advance in this direction may change how we understand the important reciprocal host-microbiome interactions that affect the function and health of their hosts. Understanding MSW may transform how we understand the plant microbiome (Lindow and Leveau, 2002; Vorholt, 2012; Berg et al., 2014; Vacher et al., 2016), which would impact bio-control and agricultural practices. It also has implications for human public health, as MSW prevalently occurs on our surfaces at home, work, food processing facilities, and hospitals. In summary, studies of MSW as a microbial habitat will have broad implications on our understanding of microbial life on diverse terrestrial microbial habitats, potentially leading to future benefits for human health, agriculture, and nature conservation.

## Data Availability Statement

The original contributions presented in the study are included in the article/supplementary material, further inquiries can be directed to the corresponding author.

## Author Contributions

All authors listed have made a substantial, direct and intellectual contribution to the work, and approved it for publication.

### Conflict of Interest

The authors declare that the research was conducted in the absence of any commercial or financial relationships that could be construed as a potential conflict of interest.
